# Factors Associated with Reduced Quality of Life in Polio Survivors in Korea

**DOI:** 10.1371/journal.pone.0130448

**Published:** 2015-06-29

**Authors:** Eun Joo Yang, Seung Yeol Lee, Keewon Kim, Se Hee Jung, Soong-Nang Jang, Soo Jeong Han, Wan-Ho Kim, Jae-Young Lim

**Affiliations:** 1 Department of Rehabilitation Medicine, Seoul National University Bundang Hospital, Seongnam, Republic of Korea; 2 Department of Physical Medicine and Rehabilitation, Soonchunhyang University Bucheon Hospital, Gyeonggi, Republic of Korea; 3 Department of Rehabilitation Medicine, Seoul National University Hospital, Seoul, Republic of Korea; 4 Department of Rehabilitation Medicine, Seoul National University Boramae Medical Center, Seoul, Republic of Korea; 5 Red Cross College of Nursing, Chung-Ang University, Seoul, Republic of Korea; 6 Department of Rehabilitation Medicine, Ewha Womans University Medical Center Seoul, Republic of Korea; 7 Department of Rehabilitation Medicine, National Rehabilitation Center, Seoul, Republic of Korea; Xi'an Jiaotong University School of Medicine, CHINA

## Abstract

The purpose of this study is to assess health-related quality of life in polio survivors (PS) compared with that in the general population in Korea. Polio survivors (*n* = 120) from outpatient clinics at two hospitals, healthy controls (HC, *n* = 121) and members of the general population with activity limitations (AL, *n* = 121) recruited through a proportional-allocation, systematic sampling strategy from the Fourth Korean National Health and Nutrition Examination Survey were surveyed with self-rated health-related quality of life (Euro QoL five-dimensions). The proportion of participants who reported problems in mobility, usual activity, and symptoms of anxiety/depression were higher in the PS group compared with the HC and AL groups. There was no significant difference in the self-care dimension across the groups. Polio-specific questionnaire, pain, depression, fatigue, Modified Barthel Index (K-MBI) and Short Physical Performance Battery (SPPB) were assessed in the PS group. Those with post-poliomyelitis syndrome had greater problems in mobility, usual activity, and depression/anxiety. Polio survivors, especially those with more pain and fatigue symptoms, and those who did not have access to medical services had poorer health-related quality of life. These findings afford useful information for potential intervention improving quality of life in polio survivors.

## Introduction

Quality of life (QoL) is as important to polio survivors as is their expected life span and other survival related issues [1]. Polio-related symptoms and disabilities such as fatigue [2], depression, comorbidities [3], pain, and weakness [4] are likely explanations for reduced health-related quality of life (HRQoL). Some factors affecting quality of life, such as family, are the same for polio survivors and age-matched controls [5]; however, there may be some specific issues pertinent only to polio survivors,

In Korea, the worst polio epidemic occurred in the 1950s and 1960s, more than 10 years after those in most Western countries. Currently, the population of polio survivors in Korea is estimated at about 60,000 people, which is greater than in other Asian countries [6] and more than twice that of Japan [7]. Therefore, the population of polio survivors in Korea may have different characteristics from those observed in developed countries where previous research has focused [8, 9]. Additionally, cross-cultural differences may also influence one’s interpretation of the illness and its impact on HRQoL [10–12].

Reduced physical activity [13] and reduced participation in social activities may be related to reduced HRQoL [14]. Therefore, comparisons of HRQoL between polio survivors and the general population and between polio survivors and those with non-polio related activity limitations in the general population could help to identify the special needs of polio survivors and inform allocation of appropriate healthcare resources [15–17]. The comparison could provide greater insight into the altered HRQoL of polio survivors and give health care providers HRQoL target levels.

The purpose of this study is to compare HRQoL in polio survivors with the general population and a population with non-polio related activity limitations and to find the factors related with HRQoL in polio survivors in Korea.

## Materials and Methods

### Study population

A multi-center survey was conducted from February to December 2012. Study centers were the Seoul National University Bundang Hospital, National Rehabilitation Center, Ewha Woman’s University Hospital (EWUH), Hwaseong City Health Center (HCHC), and Jeongnip Welfare Center.

Sample size calculation was performed using published characteristics of the Nottingham EADL[18]. The expected incidence of post-polio syndrome is at least 50% and test against a contrast is 20%. We estimated that a sample of 120 patients would have 90% power to detect odds ratio (OR) 2.0 using a significance level of 5%.

**Fig 1:**
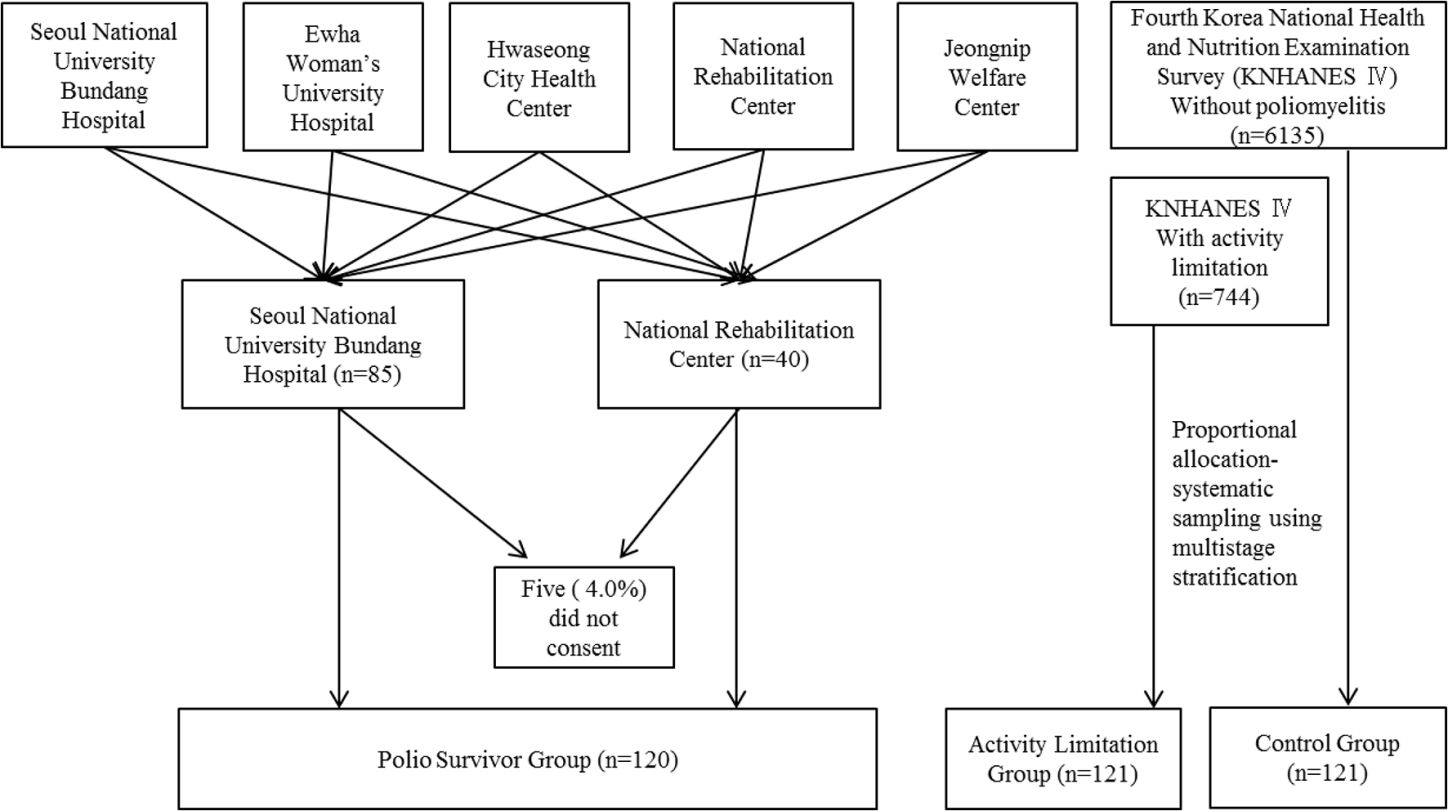
Schematic representation of data collection for the study. Outpatient Clinic at Seoul National University Bundang Hospital and at National Rehabilitation Center. K-MBI = Korean version of Modified Barthel Index; SPPB = Short Physical Performance Battery; BPI = Brief Pain Inventory; BFI = Brief Fatigue Inventory; BDI = Beck Depression Inventory; EQ-5D = Euro QoL Questionnaire 5-Dimensional Classification

We prospectively identified and invited 125 people previously diagnosed with poliomyelitis or poliomyelitis with post-polio sequelae to participate in the study as the polio survivor (PS) group. We explained that we wanted to establish a middle-aged cohort to study the late effects of polio on survivors. Prior to study consent, past medical history of poliomyelitis infection was confirmed. After subjects consented to participation, we evaluated them in two outpatient clinics: Seoul National University Bundang Hospital (*n* = 85) and the National Rehabilitation Center (*n* = 40) (Fig 1).

To compare the quality of life of polio survivors with the general population, we obtained data from the fourth Korea National Health and Nutrition Examination Survey (KNHANES IV) conducted by the Korea Center for Disease Control and Prevention. The survey included pertinent data on the population’s health, health-related knowledge and behavior, disease prevalence rate, and nutrition levels. The KNHANES was first conducted in 1998, and the fifth survey is currently underway. The participants were chosen using a proportional-allocation, systematic sampling strategy, with the population stratified for age, sex, and region. Trained interviewers conducted the structured face-to-face questionnaire to obtain socio-demographic data including age, sex, marital status, alcohol consumption, and activity limitations.

Of the 8290 survey respondents who were never diagnosed with poliomyelitis, 121 (47 men, 74 women) age- and sex-matched participants were selected for the healthy control group (HC). One hundred twenty-one (47 men, 74 women) age- and sex-matched survey respondents with non-polio related activity limitations were selected for the activity limitations group (AL). The overall mean age (range) of the three groups was 50.20 ± 7.75 (27–80) years (Fig 1).

### Ethics Statement

The protocol of this study complies with the Declaration of Helsinki. All participants were fully informed regarding study participation and provided written informed consent. The study including this consent procedure was approved by the institutional review board of Seoul National University Bundang hospital (IRB No: B-1112-142-003).

### Assessment

HRQoL and demographic characteristics were assessed in the PS, HC and AL group. Polio-specific questionnaire, pain, depression, fatigue, Modified Barthel Index (K-MBI), Short Physical Performance Battery (SPPB) were assessed only in the PS group.

#### Quality of life (QoL)

Health-related QoL was assessed using the Euro Quality of Life Questionnaire 5-Dimensional Classification (EQ-5D), which evaluates QoL in terms of five dimensions: mobility, self-care, usual activity, pain/discomfort, and anxiety/depression [19]. The responses to the EQ-5D (no problems, some or moderate problems, and extreme problems or unable to function) were collapsed into two categories (no problem and any problems (some or moderate or extreme problem or unable to function)). Using a combination of these items, a single health index score was calculated using the Korean valuation set developed by the Korean Centers for Disease Control and Prevention [20].

#### Polio-specific questionnaire

The polio-specific questionnaire contained detailed questions about the age of onset of poliomyelitis, the presence of upper and/or lower limb(s) paralysis, the presence of gradual or sudden onset of progressive and persistent muscle weakness /abnormal muscle fatigability, and the onset of post-polio symptoms. The interval between acute poliomyelitis and the onset of symptoms related to post-polio syndrome (PPS) and the duration of those symptoms were evaluated. Use of orthoses or walking aids, current ambulatory function, the disability rating registered in the national welfare system, self-reported health status, past medical history including surgical treatments, history of falling in the previous year, and medical comorbidities were evaluated. Information on treatment received for sequelae of poliomyelitis was also collected.

The diagnostic criteria for PPS, adopted from the European Federation of Neurological Societies (EFNS), were as follows: (1) prior paralytic poliomyelitis with evidence of motor neuron loss, as confirmed by history of acute paralytic illness, etc.; (2) a period of partial or fairly complete functional recovery after the acute paralytic poliomyelitis, followed by an interval (usually of 15 years more) of stable neurological function; (3) gradual or sudden onset of progressive and persistent muscle weakness or abnormal muscle fatigability (decreased endurance) with or without generalized fatigue, muscle atrophy, weakness in the limb(s), or muscle and joint pain; (4) symptoms persisting for at least 1 year; and (5) no other possible causes that could be producing PPS symptoms. Subjects who met all of the above criteria were categorized as belonging to the PPS group, and the others as the non-PPS group.

#### Pain

Pain was measured using the Brief Pain Inventory (BPI) [21], a validated pain measurement tool using numeric rating scales to assess both the intensity of pain (sensory dimension) and the interference with the patients' life (reactive dimension). The reliability of the BPI [22] has been demonstrated over short intervals using test–retest item correlation, with good results: *r* = 0.82 for worst pain, *r* = 0.75 for usual pain, and *r* = 0.83 for current pain.

#### Depression and fatigue

Depression was assessed with the Korean version of the Beck Depression Inventory (BDI-K) [23]. The Korean version of the Brief Fatigue Inventory (BFI-K) [24] was used to asses fatigue. The BFI consists of nine items on a single page. Fatigue and its effects are measured on a numeric scale from 0 to 10.

#### Modified Barthel Index (K-MBI)

The Korean version of Modified Barthel Index (K-MBI) [25] was used to assess functional ability.

#### Short Physical Performance Battery (SPPB)

The Short Physical Performance Battery (SPPB) of the Established Populations for the Epidemiologic Study of the Elderly was used to assess lower extremity performance [25].

#### Demographic characteristics

Demographic characteristics included age, gender, marital status (living with/without spouse), education level (less than middle school graduate/ high school graduate or higher), residential area (metropolitan area/city or country), and monthly individual income (less than US $1779.00/more than US $1779.00 per month). Individuals who were legally married or cohabiting were considered to have a spouse; single, divorced, or separated individuals were categorized as not having a spouse.

Information about various comorbidities was also collected. Hypertension was identified in individuals who met at least one of the following three criteria: physician diagnosis of hypertension, self-report of antihypertensive drug intake, and systolic blood pressure (SBP) 140 mm Hg or diastolic blood pressure (DBP) 90 mmHg. Blood pressure was measured manually twice at 30-second intervals after a minimum of 5 minutes of rest in a seated position, and the mean values were used to identify hypertensive participants. Diabetes was diagnosed in subjects with fasting plasma glucose 126 mg/dL and in patients who were identified in the health interview survey as actively using an oral hypoglycemic agent or insulin. Diagnosis of metabolic syndrome was based on the presence of three or more of the following: (1) waist circumference ≥90 cm for men or ≥80 cm for women [22]; (2) triglyceride levels ≥150 mg/dL; (3) high-density lipoprotein cholesterol levels <40 mg/dL for men or <50 mg/dL for women; (4) SBP 130 mmHg or DBP 85 mmHg or self-report of antihypertensive drug therapy; and (5) fasting plasma glucose level 100 mg/dL or self-report of ongoing treatment with an oral hypoglycemic agent or insulin. Information regarding ischemic heart disease and cerebrovascular accidents was acquired from self-reported history. Ischemic heart disease included angina pectoris and myocardial infarction.

### Statistical Analysis

Data are presented as frequencies and percentages for categorical variables. Continuous variables are reported as means with standard deviations.

Chi-squared tests were used to compare the PS, AL, and HC groups for differences in demographic, socioeconomic, and psychological factors, health-related behavioral patterns, and the proportion reporting any problems in each EQ-5D dimensions. A one-way analysis of variance was used to demonstrate the linearity of continuous variables among the PS, AL, and HC groups.

To assess the relationship between impaired health utility (any problem in each EQ-5D dimension) and clinical or demographic data in polio survivors, logistic regression analysis were performed. To analyze the determinants of problems in each of the five EQ-5D dimensions, a multivariate logistic regression analysis was performed with the presence of “any problems” as the dependent variable. Covariables that demonstrated co-linearity were excluded from the multivariate analyses.

Analysis of covariance (ANCOVA) with the Bonferroni correction was used to estimate age-adjusted distributions of the EQ-5D utility score according to groups. All analyses were conducted using SPSS software (version 19.0, SPSS, IL), and *P* < 0.05 indicated statistical significance.

## Results

### Characteristics of polio survivors, subjects with activity limitations, and the general population

Table 1 presents the distributions of participants in terms of demographic characteristics, health-related behavior, clinical status, and use of healthcare services. A greater percentage of the PS group had graduated from high school or completed further higher education (82.3%) compared with the HC (65.2%) and AL (52.15%) groups. Most subjects were married, but a greater number of polio survivors were living without a spouse (20.8%) compared with the HC (3.3%) and AL (9.9%) groups. The EQ-5D score was significantly lower in polio survivors than in the AL and HC groups.

**Table 1:** Characteristics of the polio survivors (PS), the general population, and the group with activity limitations.

Variables	Polio survivors(n = 120)	Activity limitations(*n* = 121)	General population(*n* = 121)	*P* for trend
Demographic factors				
Age (years)	50.20 ± 7.75	50.20 ± 7.75	50.20 ± 7.75	1
Gender				
Male	47 (38.8)	47 (38.8)	47 (38.8)	1
Female	74 (61.2)	74 (61.2)	74 (61.2)	
Level of education				
Middle school or less	21 (17.7)	58 (47.9)	39 (34.8)	
High school or more	98 (82.3)	63 (52.1)	73 (65.2)	
Marital status				
Married	95 (79.2)	109 (90.1)	116 (96.7)	<0.001
Living without spouse	25 (20.8)	12 (9.9)	4 (3.3)	
Income ($)^1^				
<1779	70 (58.3)	73 (60.3)	60 (50.4)	
≥1779	50 (41.7)	48 (39.7)	59 (49.6)	
Residence				
Metropolitan area	68 (56.2)	59 (48.8)	61 (50.4)	
City or country	53 (43.8)	62 (51.2)	60 (49.6)	
Health behaviors and clinical factors				
Comorbidity				
Hypertension	20 (30.3)	37 (30.6)	30 (26.8)	0.792
Stroke	1 (1.5)	4 (3.3)	1 (0.9)	0.401
MI / angina	0 (0)	0 (0)	3 (2.7)	0.08
Asthma	0 (0)	7 (5.8)	5 (4.5)	0.149
Diabetes myelitis	10 (15.2)	15 (12.4)	8 (7.1)	0.213
EQ-5D index (mean ± SE)	0.68 ± 0.035	0.84 ± 0.013	0.94 ± 0.015	<0.001

^1^ Income: monthly household income ($1 = 1020 KRW based on the mean exchange rate at July, 2014.

### Comparison of HRQoL of polio survivors and the general population

**Fig 2:**
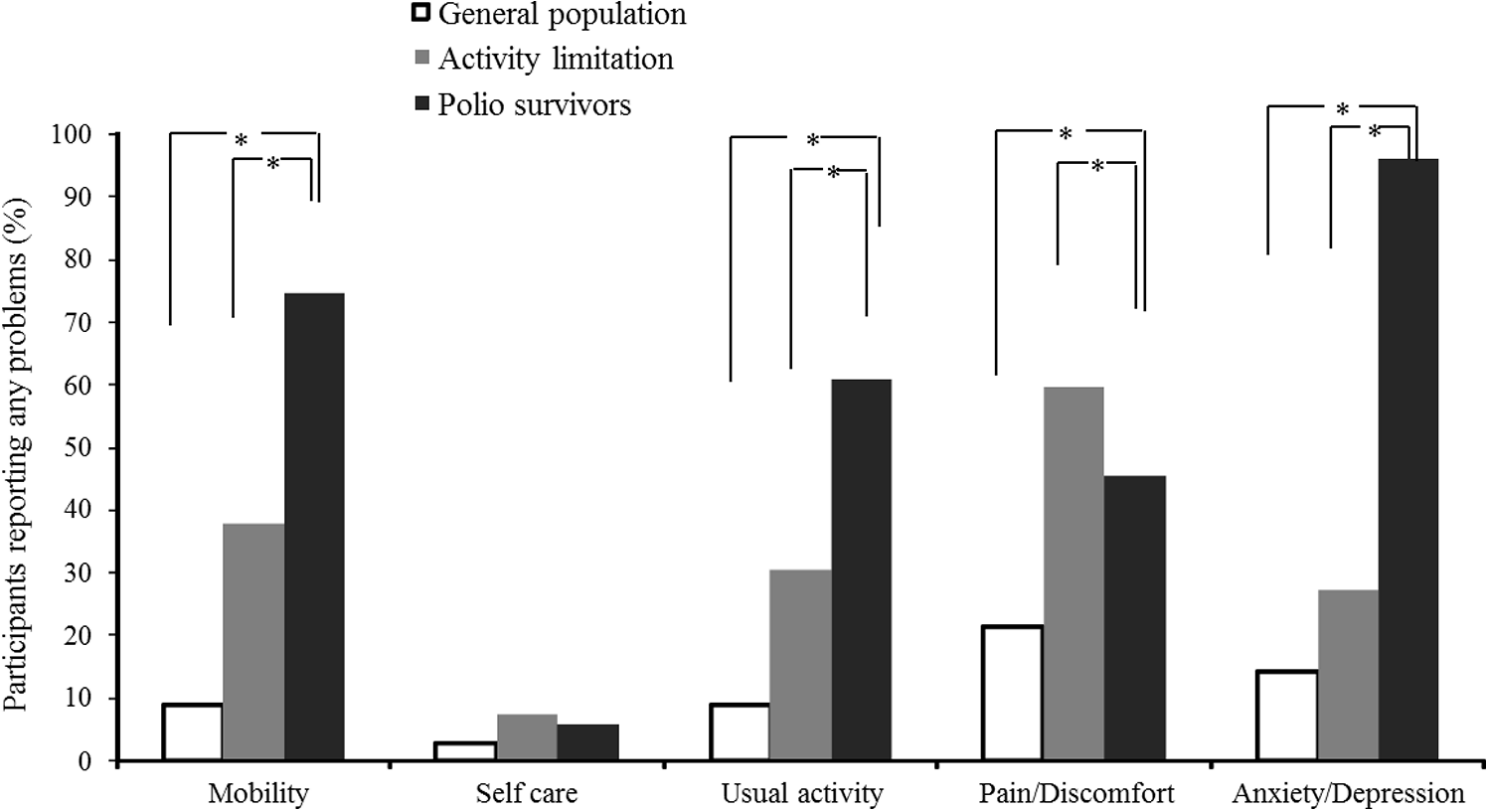
Proportion of participants who reported problems on each of the five EQ-5D dimensions.

The proportions of participants reporting problems in each dimension of the EQ-5D questionnaire are shown in Fig 2.

Compared with the HC and AL groups, the PS group reported significantly greater problems in mobility, usual activity, and the anxiety/depression dimension of the EQ-5D. There was no significant difference in the proportion of participants who reported problems in the self-care dimension among HC, AL, and PS groups. Compared with the AL group, a significantly lower proportion of participants in the PS group reported any problems related to pain/discomfort.

In the binary multivariate logistic regression analysis of EQ-5D responses, after adjusting for sex, age, education, marital status, household income, place of residence, insurance, smoking, drinking, and comorbidity (associated with QoL in previous studies), the PS group had significantly greater problems related to mobility (OR, 29.92; 95% CI, 13.85–64.64; *P* < 0.001), usual activity (OR, 15.84; 95% CI, 7.52–33.40; *P* < 0.001), and pain/discomfort (OR, 3.06; 95% CI, 1.72–5.44.64; *P* < 0.001). Compared with the activity limitations group, polio survivors had significantly higher ORs for poor self-care (OR, 4.78; 95% CI, 2.75–8.32; *P* < 0.001) and usual activities (OR, 3.53; 95% CI, 2.07–6.01; *P* < 0.001) after adjusting for the same variables.

### Factors associated with HRQoL according to the logistic regression analysis

Table 2 presents the results of the multivariate logistic regression analysis examining the predictive power of demographic characteristics, health-related behaviors, clinical status, and use of healthcare services for the EQ-5D dimensions.

**Table 2:** Logistic model for EQ-5D dimensions of polio survivors (n = 120).

	EQ-5D dimensions				
	Mobility	Self-care	Usual activity	Pain/discomfort	Anxiety/depression
Demographic factors					
Gender (female)	0.75 (0.34–1.68)	1.12 (0.24–5.22)	0.44 (0.21–0.93)*	0.20 (0.08–0.50)**	0.44 (0.21–0.92)*
Age	1.02 (0.96–1.08)	1.01 (0.97–1.06)	1.01 (0.96–1.05)	1.02 (0.96–1.08)	1.01 (0.97–1.06)
(50, <60)	1.63 (0.68–3.88)	4.00 (0.43–36.98)	1.56 (0.72–3.37)	0.80 (0.34–1.91)	1.05 (0.49–2.23)
(60)	1.78 (0.34–9.33)	5.89 (0.34–102.88)	0.80 (0.21–3.09)	3.67 (0.85–15.77)	1.21 (0.31–4.67)
Education (more than high school)	1.77 (0.48–6.54)	0.40 (0.22–0.73)*	2.47 (0.84–7.26)	1.77 (0.48–6.54)	1.32 (0.51–3.37)
Marital status (other ^a^)	0.81 (0.29–2.24)	053 (0.23–1.21)	0.65 (0.27–1.53)	0.81 (0.29–2.24)	0.53 (0.23–1.21)
Income ^b^	1.06 (0.46–2.43)	0.00 (0.00–0.01)	1.48 (0.70–3.16)	1.02 (0.42–2.47)	1.46 (0.69–3.03)
Residence (metropolitan)	1.60 (0.65–3.94)	0.20 (0.02–1.73)	0.79 (0.38–1.64)	1.60 (0.65–3.94)	0.79 (0.38–1.62)
Occupation	2.00 (0.82–4.90)	1.59 (0.34–7.43)	1.45 (0.69–3.02)	2.00 (0.82–4.90)	1.01 (0.49–2.07)
Health behaviors and clinical factors					
Comorbidity (yes)	0.71 (0.18–2.71)	0.00 (0.00–0.01)	0.89 (0.33–2.36)	0.71 (0.18–2.71)	1.09 (0.42–2.85)
PPS (yes)	2.62 (1.16–5.94)*	0.10 (0.01–0.81)*	2.76 (1.32–5.78)*	1.89 (0.80–4.47)	2.12 (1.03–4.39)*
Duration	1.02 (0.98–1.07)	1.02 (0.95–1.09)	1.04 (1.00–1.08)*	1.02 (0.98–1.07)	1.01 (0.98–1.05)
Orthosis	1.67 (0.69–4.00)	1.56 (0.33–7.26)	6.17 (2.72–14.00)**	1.67 (0.69–4.00)	2.02 (0.99–4.14)
Fall history	1.69 (0.64–4.44)	1.84 (0.21–15.97)	2.58 (1.09–6.11)*	1.69 (0.64–4.44)	1.80 (0.75–4.31)
Untreated	5.20 (2.03–13.31)**	0.83 (0.18–3.87)	1.56 (0.75–3.29)	5.20 (2.03–13.31)**	1.25 (0.59–2.59)
Fatigue	1.39 (1.11–1.76)*	1.20 (0.86–1.67)	1.29 (1.08–1.55)*	1.40 (1.11–1.76)*	1.47 (1.21–1.78)**
Pain	1.79 (1.37–2.35)**	1.17 (0.87–1.57)	1.23 (1.05–1.45)*	1.79 (1.37–2.35)**	1.45 (1.21–1.73)**
Depression (BDI)	1.06 (0.99–1.13)	1.08 (0.99–1.16)	1.11 (1.05–1.18)**	1.19 (1.08–1.30)**	1.23 (1.14–1.34)**
BMI	1.12 (0.94–1.34)	0.62 (0.27–1.40)	0.96 (0.84–1.09)	1.23 (0.99–1.52)	0.91 (0.79–1.04)
Grip strength	0.98 (0.95–1.00)	1.00 (0.90–1.10)	0.98 (0.96–1.01)	0.98 (0.95–1.00)	0.98 (0.95–1.00)
SPPB	1.05 (0.91–1.21)	0.00 (0.00–0.01)	0.86 (0.75–0.97)*	1.05 (0.91–1.21)	1.01 (0.91–1.13)
Metabolic syndrome	0.24 (0.03–2.10)	0.00 (0.00–0.01)	1.88 (0.63–5.59)	0.24 (0.03–2.10)	2.27 (0.73–7.08)

*P < 0.05;

**P < 0.001

Values are odds ratios (95% confidence intervals) after adjusting for age, education, income, region, comorbidity.

PS = polio survivor group; CG = control group; AL = activity limitation group.

Sex, male (reference), female; age, <50 years (reference), 50 ≤ age <60, ≥60 years; education, middle school or less (reference), high school or more; marital status, married (reference), other; income, lower 50% (reference), upper 50%; place of residence, city or country (reference), metropolitan area; smoking, non-smoker (reference), smoker; drinking, non-drinker (reference), drinker; comorbidity, no comorbidity (reference), comorbidity.

^a^ Includes single, living separately, divorced, and separated by death.

^b^ Income: monthly household income ($1 = 1020 KRW, based on the mean exchange rate at July, 2014.

Each dimension of the EQ5D scale served as a dependent variable; responses were grouped into two categories: no problem and problem.

Being female was strongly associated with fewer reported problems on dimensions of mobility (OR, 0.20; 95% CI, 0.08–0.50), self-care (OR, 0.44; 95% CI, 0.21–0.92), usual activity (OR, 0.20; 95% CI, 0.08–0.50), pain/discomfort (OR, 0.20; 95% CI, 0.08–0.50), and anxiety/depression (OR, 0.44; 95% CI, 0.21–0.92) dimensions.

PPS was strongly associated with restricted mobility (OR, 2.62; 95% CI, 1.16–5.94), activity limitations (OR, 2.76; 95% CI, 1.32–5.78), and greater anxiety or depression (OR, 2.12; 95% CI, 1.03–4.39), but fewer problems with self-care (OR, 0.10; 95% CI, 0.01–0.81).

Restricted mobility was also significantly associated with lack of treatment (OR, 5.20; 95% C, 2.03–13.31), fatigue (OR, 1.39; 95% CI, 1.11–1.76), and pain (OR, 1.79, 95% CI, 1.37–2.35).

People with high school or higher education had fewer problems with self-care (OR, 0.40; 95% CI, 0.22–0.73) than did those with less education.

Greater pain and discomfort were associated with lack of treatment (OR, 5.20; 95% CI, 2.03–13.31), fatigue (OR, 1.40; 95% CI, 1.11–1.76), pain (OR, 1.79; 95% CI, 1.37–2.35), and depression (OR, 1.19; 95% CI, 1.08–1.30).

Fatigue (OR, 1.47; 95% CI, 1.21–1.78), pain (OR, 1.45; 95% CI, 1.21–1.73), and depression (OR, 1.23; 95% CI, 1.14–1.34) were also significantly associated with the anxiety/depression dimension.

## Discussion

We set out to compare the HRQoL of polio survivors with a sample of the general population and a population with non-polio related activity limitations and to clarify factors that contributed to the HRQoLin polio survivors. Survivors of polio had significantly lower a HRQoL, particularly in the domains of mobility, usual activities, pain/discomfort and anxiety/depression. Survivors of polio who did not receive treatment for the sequelae of poliomyelitis and who had more pain and fatigue symptoms reported lower HRQoL.

In the current study, the HRQoL of polio survivors was shown to deteriorate more than that of a population with activity limitations; this finding may affect decisions about public health or social welfare policies. It is well known that new symptoms associated with PSS negatively affect functional status and lead to decreased satisfaction, with the greatest impact on mobility-related activities [4, 26–28]. Kling et al. [4] compared patients suffering from the late effects of polio (post-polio) to the general population. Their study found that the patients with post-polio had significantly poorer functional status and HRQoL than the general population. On et al. [2] found that polio survivors not only with PPS but also without PPS had lower HRQoL than normal control. Among stable-functioning polio survivors, health problems concerning mainly physical mobility and activity limitations were the major contributing factors for poor QoL. Restrictions in mobility and in activities had a greater effect on HRQoL in polio survivors than on the general public, suggesting that polio survivors have a particularly strong need for continuous management and support.

Although a diagnosis of PPS did not significantly reduce QoL, analyses of the sub-dimensions showed that polio survivors with PPS had more restricted mobility, greater activity limitation, and greater depression/anxiety.

Numerous factors such as gender, occupation, fatigue, pain, depressive symptoms, orthotic use, and having unmet health care needs influence QoL. One study demonstrated that new muscle weakness or disability only partly explains impaired QoL [29]. Factors other than neurological disability, such as fatigue, seem to play a role in the QoL of patients with post-polio syndrome [2]. The greater impact on QoL in patients with PPS seems not to be attributable to decreased muscle strength [2]. Strength itself explained only 14% of the physical mobility in patient with PPS [29]. In our study, physical performance was correlated with HRQoL, especially in the dimension of usual activity. Pain was found to be a prominent symptom in other studies, and it was associated with poorer HRQoL [30, 31]. Polio survivors who did not receive treatment of the sequelae of poliomyelitis had lower HRQoL. People with disabilities need general medical services for the treatment of current disabilities [32]. Although a previous study found that perceived treatment for activity limitations was correlated with socioeconomic level [33], we found the unmet health care needs were associated with poorer HRQoL even after adjusting for socioeconomic factors. In particular, people with unmet healthcare needs had poorer mobility and greater pain/discomfort.

In contrast to the differences regarding physical abilities, no significant differences concerning the area of self-care were found among the groups. A recent study found that satisfaction with one’s relationship with a partner and with family life among post-polio patients did not seem to be affected by the new deteriorating condition [4].

A previous study found that age and number of body parts affected by polio were associated with physical and functional status [4]. Age has previously been reported to be associated with the presence of post-polio symptoms [34, 35]. However, age was not significantly associated with poorer HRQoL in the present study. The reason for this is unclear, but the patients in this study were mainly aged from 40 to 50 years, and further symptoms may develop as they age [36]. A prospective study would be needed to fully evaluate changes in HRQoL related to aging in polio survivors.

Our study has some limitations. We used a sub-optimal sampling method, as we could not randomly sample or statistically assess the representativeness of our sample. Due to the cross-sectional design of our study, we were unable to determine causal relationships. Nevertheless, our study did compare the HRQoL of polio survivors with that of the general population and examined the patients’ QoL. Therefore, findings from this study provide a general comparison of the HRQoL of polio survivors and the general public. In addition, there were no data in the general population such as BPI, BFI, SPPB, and variables in the polio specific questionnaire, so we could not compare the same factors related with HRQoL in other group.

In conclusion, the QoL of polio survivors is worse than that of the general population in terms of physical and functional status. Polio survivors experienced greater restriction in mobility and activity, greater pain, and greater depression/anxiety compared with controls, although there was no difference in the self-care domain. To improve the HRQoL of polio survivors, we need to provide better rehabilitation services.
